# Political ecology of climate change adaptation in the Arctic: Insights from Nunatsiavut, Canada

**DOI:** 10.1057/s41599-025-06058-2

**Published:** 2025-11-20

**Authors:** Ishfaq Hussain Malik, James D. Ford, Robert G. Way, Nicholas E. Barrand

**Affiliations:** 1 University of Leeds, Leeds, UK; 2 Priestley Centre for Climate Futures, University of Leeds, UK; 3 Queen’s University, Kingston, Canada; 4 University of Birmingham, Birmingham, UK

**Keywords:** Environmental studies, Geography

## Abstract

Political ecology analyses climate change adaptation by examining the intricate relationships between systemic inequalities, power dynamics, and structural factors, including colonialism and capitalism. This paper examines the political ecology of climate change adaptation in the Arctic, focusing on five Inuit communities in Nunatsiavut, a self-governing Inuit region in northern Canada. It examines how various social, economic, and environmental factors intersect to influence adaptation. We found that colonialism, forced relocation, and capitalism are driving the historical construction of climate risk along with contemporary adaptation challenges, and showcase how inequities affect the ways different community members experience and respond to climate change. Inuit communities face significant adaptation barriers, such as high costs associated with store-bought food and machinery, economic constraints, and technological dependence required for food gathering. Using a political ecology lens, we contextualised these barriers within the broader socioeconomic factors. The analysis centres on the critical question of “adaptation for whom?” and examines the barriers and limits to adaptation, emphasising the uneven distribution of adaptive capacity within Nunatsiavut. This study underscores the need for an equitable approach to adaptation that addresses the systemic, structural, and infrastructural challenges faced by Inuit in a rapidly changing Arctic. This research was conducted in accordance with Indigenous and Inuit research ethics, ensuring Inuit self-determination and community control over the research process.

## Introduction

The Arctic is at the nexus of capitalism, neocolonialism, neoliberal development, geopolitics, and climate change, making it a frontline in the global struggle against environmental change. Understanding this nexus is important for analysing climate change impacts, adaptation responses, and the political ecology of how climate change creates social, economic, and environmental injustices. Political ecology herein refers to the study of intricate relationships between political, economic, and social factors to analyse environmental issues, recognising that they are deeply entwined with structural factors, power dynamics, economic interests, and historical processes (Robbins 2019; Malik 2024). Climate change adaptation intersects with political ecology challenges and pressures (Sovacool 2018a) and is inherently a political ecology problem (Scoville-Simonds et al. 2020). Challenges to adaptation often originate from unequal distribution of power and resources, exacerbating existing social and economic inequalities and environmental injustices (Rice et al. 2022; Amorim-Maia et al. 2022).

Climate adaptation involves adjustments in ecological, social, or institutional systems in response to actual or expected climate impacts and are aimed at reducing harm or taking advantage of beneficial opportunities (IPCC 2023). The process and conceptualisation of climate change adaptation extend beyond technical fixes and are deeply intertwined with economic, social, and political dynamics (Eriksen et al. 2015), essentialising a political ecology understanding of adaptation. Yet dominant framings of adaptation typically overlook the contribution of political and economic systems to climate change and its impacts (Lindegaard 2018; Malik and Ford 2025a). These framings focus on technical solutions such as early-warning systems, sea walls, carbon capture and storage, engineered permafrost protection, climate services, and climate-resilient infrastructure (IPCC 2023; Steele et al. 2023) without addressing the systemic and underlying drivers of risk. Consequently, adaptation strategies may reinforce existing inequalities and fail to challenge the underlying power structures, social injustice, and economic inequalities that perpetuate environmental degradation (Schipper et al. 2021; 2020a; Tschakert et al. 2023; Malik and Hashmi 2022; Singh 2024). By overlooking these root causes, such understandings of adaptation may promote or support interventions which inadvertently reinforce or create new sources of vulnerability (susceptibility to climate change), exacerbating the very problems they aim to mitigate (Schipper et al. 2020b; Rahman et al. 2023; Malik and Ford 2024; Eriksen et al. 2021; IPCC 2023). Understanding this issue through the lens of political ecology allows for a critical examination of how power relations, economic interests, and social inequalities shape environmental outcomes and adaptation strategies (Malik 2024; Shackleton et al. 2023).

Climate change is defined as long-term alterations in climate, identifiable through shifts in the average or variability of climate properties, and persisting over decades or longer (IPCC 2018). Climate change in the Arctic is a global concern due to its ecological, social, and economic implications, and colonial history. Colonialism is the historical and ongoing process through which Indigenous lands, governance systems, and knowledge were displaced and marginalised by settler governments, policies, and institutions (Fanon 2001; Lefevre 2015; Smith 2021). In the Arctic, this includes forced relocations, cultural assimilation, and structural underinvestment in Indigenous communities (Liboiron 2021; Das 2020; White 2023).

The Arctic is experiencing rapid climate change and is warming nearly four times faster than the global average (Rantanen et al. 2022; Dai et al. 2019), with potentially wide-ranging consequences for both the region and the globe (IPCC 2023). Adaptation strategies in the Arctic have largely focused on improved prediction of changes, green energy solutions, satellite monitoring of ice conditions, deploying new technologies for ice safety, and shifting infrastructure in response to thawing permafrost (Meinander et al. 2022; Malik et al. 2025a; Li et al. 2024; Ford et al. 2016), rather than understanding the socio-political roots of vulnerability. The Canadian Arctic, in particular, is a global hotspot of climate change impacts and is experiencing significant changes in sea ice, air temperature, and precipitation, affecting human communities (Schoeppner 2024; Landrum and Holland 2020). These changes are having wide-ranging implications for Indigenous Peoples, resulting in disruption of traditional food systems, food security, travel routes, and cultural activities, wildfires, increasing disaster losses, and damaging infrastructure (Hancock et al. 2022; Ford et al. 2021; Cunsolo et al. 2020). Inuit communities in the Canadian Arctic are highly susceptible to climate impacts due to dependence on natural resources for livelihood and cultural activities (Schoeppner 2024; Fawcett et al. 2018). Yet these challenges are not merely environmental; they are deeply connected with historical and contemporary socio-economic-political dynamics (Malik and Ford 2025a).

Most climate change studies in the Canadian Arctic (and more generally in the Arctic) have focused on broader environmental changes, often neglecting the broader socioeconomic and multiple stressors—such as colonial legacies, economic marginalisation, housing precarity, food insecurity, and limited access to services—that influence how communities experience and respond to climate change (Lede et al. 2021; Huntington et al. 2019). When it comes to adaptation, research has tended to focus on the environmental and technical aspects (Nicu and Fatorić 2023; Solovyeva 2024; Garbis et al. 2023), often overlooking the complex socio-political and economic dimensions that shape adaptive capacities and outcomes (Birchall and Kehler 2023). The application of political ecology is important because apolitical narratives of adaptation often fail to account for power dynamics, historical contexts, and socio-economic inequalities (Robbins 2019).

Technical and apolitical approaches commonly omit the critical dimensions of power, history, and structural inequality that shape differential exposure to climate risk (Sovacool et al. 2020; Garcia and Tschakert 2022). A political ecology lens employs a social constructivist perspective, confronts climate coloniality, and enables an understanding of how climate change vulnerability is produced through ongoing colonial histories, capitalist market dependencies, and power structures that marginalise Indigenous knowledge systems and authority (Whyte 2017; Bonds and Inwood 2016; Cederlöf and Loftus 2023; Sultana 2024). Political ecology provides valuable insights into how socioeconomic factors intersect, revealing the ways in which climate change exacerbates existing vulnerabilities and creates new forms of inequity (Robbins 2019; Rudge 2023; Ajl 2023). It links local and regional environmental degradation and changes with global dynamics, politicising environmental issues and the ways in which power dynamics at both global and local levels affect adaptation (Acheampong 2020; Rusca 2024). Political ecology analyses how Inuit subsistence hunting practices and food sharing networks are an important part of the cultural core—that is, the foundational social and economic practices that sustain Inuit identity, community cohesion, and relationships to the land (Wenzel 2013; Collings et al. 2016). However, its application is lacking in understanding climate change in the Arctic (Malik and Ford 2025a). This paper responds to this gap by using a political ecology lens to analyse how socioeconomic factors operating across multiple spatio-temporal scales shape Inuit experiences of and responses to climate change. This approach helps us interrogate key questions such as: adaptation for whom? What are the limits and barriers to adaptation, and who faces these barriers most acutely?

By foregrounding issues of environmental justice and examining who benefits from adaptation—and who does not—political ecology offers a nuanced understanding of adaptation including factors which may often be overlooked. This perspective is particularly important in the Arctic, where Indigenous Peoples are not only on the front lines of climate change but are also navigating legacies of colonialism, economic marginalisation, and environmental degradation.

Nunatsiavut, a self-governing Inuit region in Labrador, Canada, presents a compelling case for the study of political ecology of climate change adaptation in the Arctic due to its history of forced relocation, communities’ reliance on vulnerable ecosystems, and climate and socioeconomic challenges. Historically, Inuit in Nunatsiavut have experienced multiple waves of colonial disruption, including the establishment of Moravian missions, government-led centralisation policies, and forced relocations of the communities of Hebron, Okak, and Nutak (Brice-Bennett et al. 1977; 2023; Stopp 2024). These relocations severed ties to historic homelands, disrupted subsistence practices, and weakened social cohesion—effects still felt across generations (Brice-Bennett 2017; Hanrahan 2003; Kennedy 1977; Richling 1985). Today, many Inuit in Nunatsiavut rely on a hybrid economy of wage labour and subsistence hunting, fishing, and gathering (Middleton et al. 2020; Fleming et al. 2012; Fleming 2009). However, this dependence on vulnerable ecosystems is complicated by climate-driven environmental changes across cryosphere, hydrosphere and ecospheres (thinning ice, unpredictable weather, changing animal migration) and socio-economic challenges such as food insecurity, technology costs, and limited infrastructure (Nunatsiavut Government, 2025; Wang and Way 2025; Johnson et al. 2024; Bishop et al. 2025; Malik et al. 2025b). These conditions position Nunatsiavut as a critical case for exploring how adaptation is constrained and shaped by intersecting historical, political, and environmental forces.

In Nunatsiavut, adaptation planning has not sufficiently addressed the effects of forced relocations, colonial dominance, dispossession, erosion of land-based livelihoods, or economic dependence on southern markets (Nunatsiavut Government 2024; Stopp 2024). While investments in climate-resilient housing or subsidised food programmes are important, they fail to account for the cultural and relational dimensions of food sovereignty, mobility, and intergenerational knowledge transmission—factors central to Inuit adaptation practices (Nunatsiavut Government 2024; ITK 2021). This illustrates the need for a political ecology approach that centres Inuit lived experience and challenges technocratic, one-size-fits-all solutions.

This paper explores the political ecology of climate change adaptation, based on research conducted with five communities in the self-governing Inuit region of Nunatsiavut, Canada. It examines how climate change reinforces existing socio-economic inequalities and creates new adaptation challenges and risks; charts the differential impacts of climate change and associated environmental justice considerations; and assesses the key processes shaping how adaptation takes place, by and for whom, including barriers and limits to adapting.

## Research approach

### Methodological and conceptual framing

The methodology employed in this study is informed by work on decolonisation, actively including Indigenous voices and knowledge. This approach utilised a bottom-up framework, involving extensive community engagements to co-develop the approach used and identify key themes for the research. Through this participatory process, the methodology ensures that the work is grounded in a community-engaged approach and the lived experiences that prioritise Inuit perspectives (Joseph et al. 2022; Zurba et al. 2022). It ensures the application of a decolonial lens to challenge the epistemic privilege and authority in Eurocentric or Western knowledge systems (Fanon 2001). Decolonial research methodologies involve the voicing of Indigenous forms of knowledge and the representation of reality as seen through the eyes of Indigenous Peoples (Denscombe 2024). Decolonising research involves fostering what Rauna Kuokkanen, a Sami scholar, calls “multi-epistemic literacy” that promotes learning and dialogue between different epistemic worlds and an ability to read, write, listen, hear, and learn (Kuokkanen 2007; Sundberg 2014).

While this study was grounded in participatory methods, it was explicitly informed by decolonising methodologies that challenge Eurocentric epistemologies and settler-colonial research norms. Decolonising approaches shaped not only who was involved in the research, but how knowledge was defined, shared, and discussed. Indigenous community members led the framing of research questions and interpretation of findings, centring Inuit knowledge systems, lived experiences, and relational ethics. Prioritising Indigenous ways of knowing and Indigenous voices inform our cultural and ethical practices and conversations about how Indigenous knowledge is understood, explained, and used (Jacobs 2025). Importantly, we do not conflate decolonising methods with community engagement alone, recognising that not all participatory work is inherently decolonial (Tuck and Yang 2012). Rather than extractive or symbolic inclusion, this study prioritised Inuit governance, accountability, and documentation of local adaptation strategies and challenges. Challenging Eurocentric authority also meant creating space for Inuit stories and strategies as primary sources of expertise, rather than supplementing dominant scientific narratives.

This study was designed in a respectful and decolonial manner, addressing and incorporating community concerns and priorities while avoiding extractive knowledge practices. These decolonial methods foster reciprocal relationships between researchers and the communities they study, which is crucial because traditional research methods often reinforce colonial power dynamics, scholarship, imagery and bureaucracies by appropriating Indigenous knowledge rather than promoting knowledge exchange and community priorities (Said 1978; Omodan 2025; Zurba and Papadopoulos 2023; Joseph et al, 2022). Linda Tuhiwai Smith (2021) offers a critical analysis of the colonial foundations of research, emphasising that it has historically functioned as a tool of imperial domination, deeply entangled with the classification, representation, and control of Indigenous Peoples through Western epistemologies. In many Indigenous contexts, the term ‘research’ evokes distrust and trauma, recalling extractive practices that have devalued Indigenous knowledge systems and experiences (Smith 2021). This critique underscores the need for decolonial approaches that reorient research as a practice of relational accountability, reciprocity, and community benefit—particularly within Inuit and other Indigenous contexts. In this study, these principles were central to ensuring that research was conducted with communities, not on them, supporting Inuit self-determination and knowledge sovereignty.

We draw upon political ecology to examine climate change adaptation in Nunatsiavut. Political ecology asserts that social and economic inequalities shape how climate change is experienced and responded to (Cederlöf and Loftus 2023). It recognises that climate change is not just a scientific issue but also a deeply political and social one (Tornel 2023). Technology and wage economy play an important role in the transformation of subsistence practices, food security, economic arrangements, and livelihoods (culture core) of Inuit in the Arctic (Harder and Wenzel 2012; Wenzel 2009; Collings 2011). This is important in understanding the relationship between climate change and Arctic peoples.

By focusing on Inuit communities in Nunatsiavut, political ecology is useful in understanding how climate change, environmental justice, and multiple dimensions of socio-economic inequalities intersect, influencing not only how adaptation occurs but also who benefits from it (Rusca 2024; Kleinod-Freudenberg 2024). It asserts that disparities in adaptation are caused by socio-economic factors (Walker et al. 2024). Political ecology provides a critical, wider political-economic lens to examine how power relations and structural inequalities, rooted in broader historical and economic contexts, shape the adaptive capacities of communities (Harcourt et al. 2023; Barca et al. 2023).

Political ecology contends that a person’s agency and access to resources are affected by multiple intersecting social constructs, such as class, gender, and race, and that material inequalities are normalised through dominant constructs to make them appear as self-evident and natural (Cederlöf and Loftus 2023; Sundberg 2016). Political ecology demonstrates that power is embedded in knowledge systems, revealing that power and knowledge co-produce one another, and that dominant European epistemologies systematically marginalise non-European and Indigenous ways of knowing at resource frontiers—reinforcing global hierarchies rooted in colonial, racial, and class-based inequalities (Quijano 2000; Smith 2021; Mignolo 2013).

### Study area

**Fig. 1:**
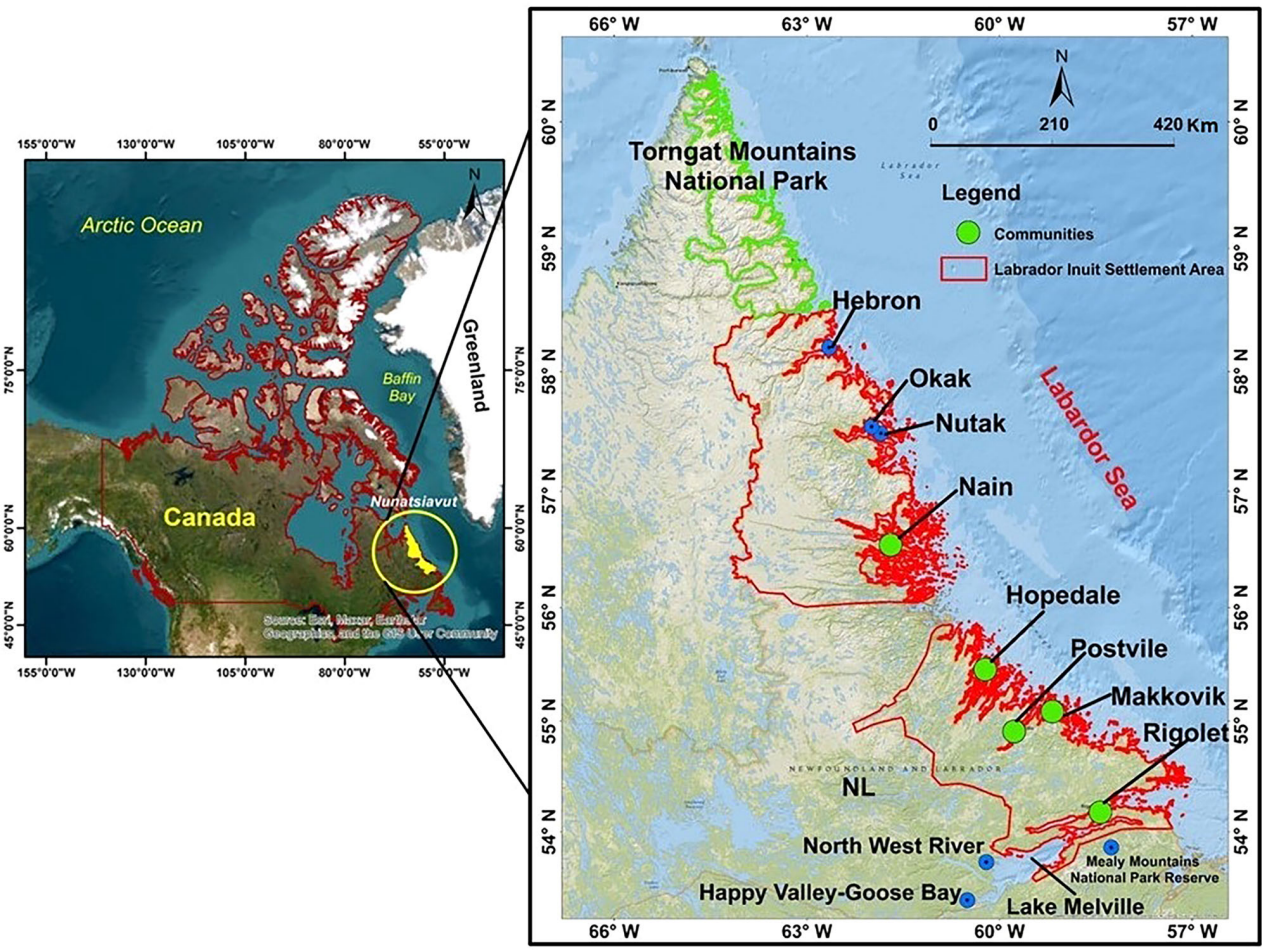
Map showing the location of the five communities of Nunatsiavut from north to south (Nain, Hopedale, Makkovik, Postville, and Rigolet), LISA excluding the zone, three relocated communities of Nutak, Okak, and Hebron, Torngat Mountains National Park, Mealy Mountains National Park Reserve, and Happy Valley-Goose Bay.

This research was conducted with all five Inuit communities of Nunatsiavut—Nain, Hopedale, Makkovik, Postville, and Rigolet (Fig. 1 and Table 1)—located between 54° 10′ N, 58° 26′ W (Rigolet, the southernmost community) and 56° 54′ N, 61° 69′ W (Nain, the northernmost community). Nunatsiavut, meaning “Our Beautiful Land,” is one of the four Inuit regions of Inuit Nunangat—the Inuit homelands of northern Canada, located on the eastern coast of northern Labrador. Inuit in Nunatsiavut speak Inuktitut/Inuttitut and/or English. People from Nunatsiavut are called Nunatsiavummiut. Nunatsiavut is the first Inuit region in Canada to achieve formal self-governance (Labrador Inuit Association 2005). The land claims agreement holds significance for Indigenous sovereignty and serves as a settlement regarding the extent of Aboriginal rights for the Labrador Inuit, encompassing both governance, land ownership rights, and resource management (White, 2023). Nunatsiavut consists of Labrador Inuit Settlement Area (LISA), including 72,520 sq. km of land and 44,030 sq. km of sea known as the Zone, five communities with a population of 2095, Labrador Inuit Lands (LIL) with ownership rights of Inuit comprising of 15,800 sq. km, lands where Inuit have special harvesting rights (Schedule 12-D), and Torngat Mountains National Park (Nunatsiavut Government 2019; Labrador Inuit Association 2005; Statistics Canada 2021). Of the approximately 7200 Nunatsiavummiut, about one-third live in LISA, one-third in the Upper Lake Melville area of Labrador in the communities of Happy Valley–Goose Bay, North West River, and Mud Lake, and the remaining live in other parts of Canada or elsewhere (Broomfield 2023; White 2023; Natcher et al. 2012).

**Table 1:** Characteristics of five Inuit communities of Nunatsiavut.

Community	Nain	Hopedale	Makkovik	Postville	Rigolet
Nature of community	Inuttitut name of Nain is Nunainguk. Nain is the largest and northernmost of the five communities in Nunatsiavut, as well as the northernmost town in Newfoundland and Labrador. It is the administrative capital of Nunatsiavut and was established in 1771. Nunatsiavut Research Centre is in Nain and coordinates research activities.	Inuttitut name of Hopedale is Agvitok and was earlier known as Hoffenthal. Hopedale is the second largest of the five communities. It is the legislative capital of Nunatsiavut and was established in 1782. Some of the forced relocatees from Nutak and Hebron settled in Hopedale in the 1950s.	Inuttitut name of Makkovik is Maggovik. Makkovik is the third largest and easternmost community of the five communities and was established in 1896. Fifteen families of forced relocatees from Hebron (Hebronimiut) settled in 12 houses in Makkovik in 1959 on the site known as ‘Hebron end’.	Inuttitut name of Postville is KipukKak. Postville is the smallest of the five communities in Nunatsiavut. It is located in Kaipokok Bay and between Makkovik and Rigolet, and was established in 1941. It is historically known to have important trading posts.	Inuttitut name of Rigolet is kikiak. Rigolet is the southernmost Inuit community in Inuit Nunangat. It is the southernmost and fourth largest community of the five communities in Nunatsiavut and was established in 1773.

In the 1950s, the provincial Government of Newfoundland and Labrador forcibly relocated northern Labrador Inuit communities such as Hebron and Nutak to southern settlements (Brice-Bennett et al. 1977). Okak had been abandoned earlier following the devastating impact of the 1918 Spanish Flu pandemic, which killed most of its residents and marked a profound colonial trauma for northern Labrador Inuit (Budgell 2019). The relocations were done under the pretence of improving access to healthcare and education, but they dismantled familial networks, disrupted land-based livelihoods, and were carried out without consultation or consent (Brice-Bennett et al. 1977; Natcher et al. 2012). Most of the community members from the northern settlements moved to Nunatsiavut and the Upper Lake Melville area, where communities lived at Happy Valley-Goose Bay, North West River, Sheshatshiu, and Mud Lake (Natcher et al. 2012). There are as many Nunatsiavummiut in Upper Lake Melville as there are in all of Nunatsiavut itself (Brice-Bennett et al. 2023). The relocations led to food accessibility challenges in receiving communities, deep psychological trauma, and long-term socioeconomic dislocation (Nunatsiavut Government 2019; Natcher et al. 2012). This caused dispossession, identity and language loss, and cultural rupture, whose legacy continues to shape land access, food security, and intergenerational well-being (MacDonald 2015; Stopp 2024; Procter 2020; Truth and Reconciliation Commission of Canada 2015).

Inuit in Nunatsiavut have experienced significant socio-economic and political changes, such as colonialism, hunting restrictions, mechanisation of the fishery, forced relocations, and the entrenchment of wage labour, creating dependencies on southern markets and eroding the sustainability of traditional subsistence practices (Brice-Bennett et al. 1977; Natcher et al. 2012; Cadman et al. 2023). The militarisation of the economy through the construction of military bases, airstrips, supply lines, and radar stations during the Second World War and Cold War led to ecological degradation, restricted Inuit access to traditional hunting grounds, and introduced new governance mechanisms and surveillance practices (Armitage and Kennedy 1989; Whitney Lackenbauer and Farish 2007). The signing of the Labrador Inuit Land Claims Agreement in 2005 was an important event for Nunatsiavummiut, establishing the Nunatsiavut Government and the LISA to advance Inuit self-determination and territorial autonomy and celebrate and preserve culture (Nunatsiavut Government 2019; Brice-Bennett et al. 2023; White and Alcantara 2020).

These intertwined histories of displacement, political marginalisation, and ecological disruption form the foundation of contemporary adaptation challenges. They help explain why technological or infrastructure-based solutions are often insufficient—because they fail to address the relational and historical conditions that shape how Inuit access land, food, mobility, and decision-making power.

### Positionality statement

This research was conducted by a team comprising both Indigenous and non-Indigenous researchers. The authors have longstanding collaborative relationships in Inuit Nunangat, particularly within Nunatsiavut. This study was co-designed with input from Inuit community members, and one of the co-authors is a Kallunângajuk and a beneficiary of the Labrador Inuit Land Claims Agreement (Nunatsiavut), bringing direct lived experience and cultural knowledge to the analysis. We recognise the asymmetries of power and privilege that exist within research relationships, especially when engaging with Indigenous communities. Our work draws from decolonial and Indigenous research paradigms and prioritises community knowledge, accountability, and co-interpretation. The data collection process emphasised relational ethics, with iterative dialogue, local validation, and a commitment to honouring Inuit voices, concerns, priorities, and perspectives throughout the research process. We acknowledge the limitations of conducting research within Euro-Western academic structures and are committed to continual reflection on our roles, responsibilities, and positionalities. This includes critically examining how our identities influence what questions we ask, how we interpret data, and how we represent Indigenous experiences in our work (Ortenzi et al. 2025). This study followed a participatory, community-led design that centred Inuit perspectives at every stage. Community members shaped research priorities, informed data collection, and guided the interpretation of findings. By emphasising co-production, the study aimed to redress historical imbalances in Indigenous research, prioritising local voices over external academic narratives. Findings were continuously validated through regular visits and feedback from communities to ensure they reflected lived experiences, and the research maintained a focus on reciprocity, with outcomes intended to directly support local adaptation efforts and governance.

### Methods

#### Data collection

Key themes that guided data collection were co-developed with community partners (Table 2) and focused on climate change impacts, adaptation strategies adopted, who is able to adapt, and barriers and limits to adaptation. Themes were co-developed through an iterative, collaborative process involving the research team, Nunatsiavut Government Research Advisory Committee (NGRAC), and community members through community engagement sessions. These discussions helped refine categories and ensure that the themes accurately reflected local experiences, concerns, and priorities. This process helped ground the analysis in Inuit knowledge and ensured that community perspectives remained central throughout.

**Table 2:** Key themes and questions that guided the data collection.

Key themes	Example questions
Climate observations and impacts	What kinds of changes have you seen in weather conditions, temperature, snowfall, and rainfall patterns, and how are they affecting the community?
Adaptation strategies	What are you doing to adapt to the changing climate conditions?
Adaptation in access to traditional foods	Are changing weather conditions affecting access to traditional foods? If so, how are you adapting to it? Who in the community is most impacted by these changes?
Barriers and limits to adaptation	What factors affect your ability to adapt to climate change?
Equity of adaptation strategies	Who in the community is benefiting from adaptation strategies, and who is left behind?
Access to technology	Do all families in your community have access to reliable transportation technology (e.g. skidoos, trucks, and boats) for hunting and fishing?
Affordability of technology	How affordable are technologies for you and others in the community? Is the cost of buying and maintaining technology (e.g. snowmobiles, boats) affordable for most people in the community?
Cost of gas	Can you and others in the community afford the rising cost of gas needed for transportation and hunting? How does the cost of fuel affect your ability to access traditional foods or travel to important locations?
Affordability and dependence on store-bought foods	Has reliance on store-bought food increased over time due to climate change affecting access to traditional food sources? Are store-bought foods affordable for you and others in the community? Do certain groups (e.g. elders, lower-income families) in the community struggle more with the high cost of store-bought food?

### Engagement with NGRAC

The research process began with a series of meetings with the NGRAC and with researchers who had previously led successful partnerships in the region. These engagements were critical in ensuring that the research objectives were aligned with community priorities and that the research methods were culturally sensitive. During these meetings, the research goals and proposed methods were discussed with the NGRAC, and their feedback was incorporated into the final research design. This collaborative approach helped to establish trust and ensured that the research was conducted in a manner that was respectful and relevant to the Nunatsiavut communities.

### Community engagement

**Fig. 2:**
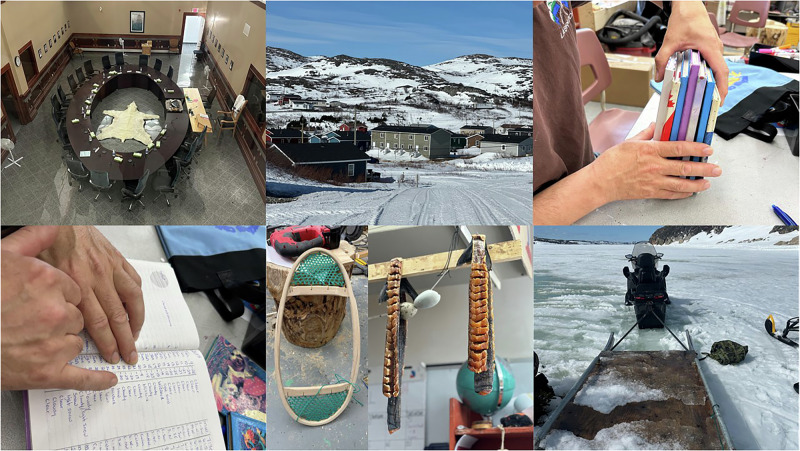
Community engagement sessions.

Following the initial consultation with the NGRAC, a comprehensive community engagement was completed in spring 2024 (Fig. 2). The purpose of this engagement process was to engage directly with rights holders, including key informants and local leaders, to identify key themes related to climate change adaptation that would guide the subsequent fieldwork. Community meetings provided an opportunity to introduce the research project and gather initial input. Informal interviews with key informants, focus group discussions, and storytelling sessions were conducted to gain deeper insights into local concerns and priorities.

Key informants were identified through a combination of community referrals and informal conversations with community members. After initial engagement, community members suggested individuals with specific local knowledge or lived experience, including Elders, hunters, teachers, and youth. In some cases, participants also self-identified during open conversations. Community meetings and storytelling sessions were promoted through local social media (community Facebook groups), and word-of-mouth via community partners. Attendance at these sessions was voluntary, and sessions were hosted in familiar, accessible community spaces with diverse genders and age groups. Informal interviews and focus groups took the form of conversational, land-based, or in-home discussions, shaped by participants’ comfort. Storytelling sessions facilitated Elders and other participants to reflect on changes over time and share intergenerational knowledge. Some sessions were shared over food, which helped foster trust, mutual respect, and reciprocity with community members. The act of sharing a meal fostered an informal and relaxed atmosphere, encouraging open dialogue and deeper engagement.

### Ethical considerations

Informed consent was obtained from all participants prior to data collection, ensuring that they were fully aware of the purpose of the study, the procedures involved, and their rights as participants. Verbal and written consent were obtained depending on participants’ preferences. Participants were made aware that they could withdraw from the study at any time without any consequences. Breaks, check-ins, and post-interview debriefs were also provided to participants. All participants were offered the choice to remain anonymous or to be identified by name, role, or community. All data were anonymised by using number codes such as Participant 1, Key informant 1, and Focus Group 1 to protect the identities of participants. Data were stored securely on encrypted, password-protected OneDrive and are accessible only to the research team and community partners, and will be stored until the end of 2025. The research design and methods were developed in consultation with NGRAC and community members to ensure they were culturally sensitive, appropriate, and respectful. Ethics approval was provided by the NGRAC (NGRAC-12770416) and the University of Leeds, UK (AREA FREC 2023-0596-660). This study is a collaborative work between Indigenous and non-Indigenous researchers, where an Indigenous researcher guided the fieldwork, themes, and research design, and non-Indigenous researchers carried out the fieldwork and research framework.

This research followed principles of data autonomy and community governance, ensuring that Inuit participants and communities retained control over how their knowledge was used, interpreted, and shared (Ortenzi et al. 2025). Community members had the opportunity to review transcripts, and findings before publication. Quotes and attributions were used only with consent, and participants could request revisions or removals at any time. No data were published that communities did not agree to share. Decisions about how findings are communicated—including whether to name specific communities or individuals—were made collaboratively with communities to respect self-determination, cultural safety, and community-defined priorities. This approach aligns with OCAP® principles (ownership, control, access, and possession) (FNIGC 1998), Tri-Council Policy Statement: Ethical Conduct for Research Involving Humans (TCPS2, Chapter 9, 2022), the United Nations Declaration on the Rights of Indigenous Peoples (UNDRIP 2007), and the National Inuit Strategy on Research (ITK 2018), emphasising Indigenous control and oversight of data related to their lives and lands.

### Main fieldwork and data collection

**Fig. 3:**
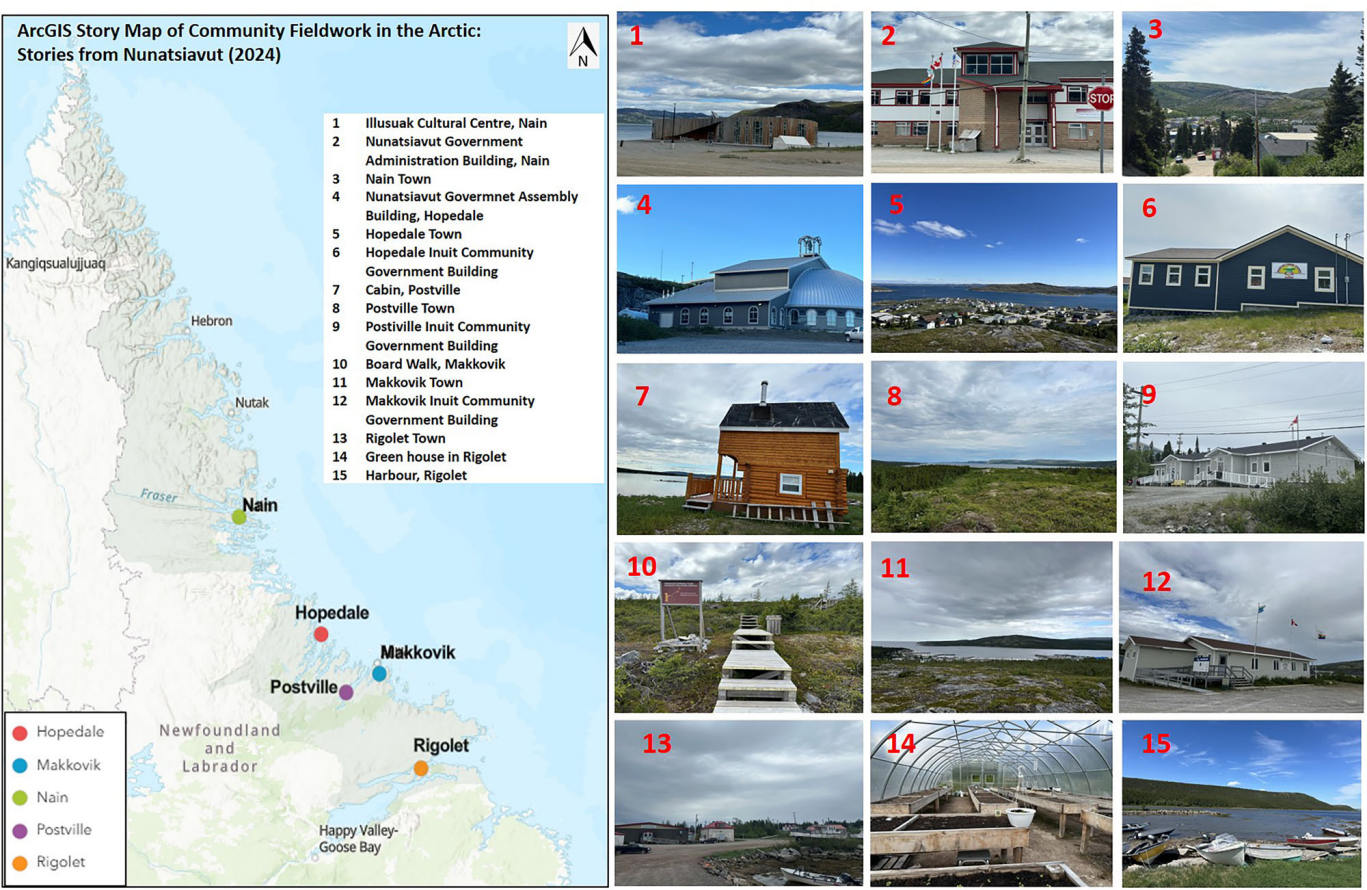
StoryMap of five Indigenous communities in Nunatsiavut, including photographs taken during fieldwork showing important features.

The main fieldwork and data collection took place in summer 2024, across five communities of Nunatsiavut (Fig. 3). Semi-structured interviews (*n* = 60) and focus group discussions (*n* = 6) were conducted with a broad cross-section of community members with diverse genders and age groups within the five communities. Key informant interviews (*n* = 14) were conducted with AngajukKâk, community leaders, and elders. These interviews provided critical insights into the specific adaptation strategies that were being implemented or considered by the communities. Interviews with community leaders were conducted to understand the leadership perspectives on governance, decision-making processes, and the broader socioeconomic and political context of climate change adaptation.

For clarity and consistency, we use the term participants to refer to all individuals involved in interviews or community engagement sessions. These included key informants, as well as youth and general community members who offered lived experiences and everyday perspectives on adaptation. When referring to individuals in the results, we retain these categories (e.g. elder, youth, and teacher) where relevant, while using participant more broadly when specific identifiers were not disclosed. Where relevant, participants are identified by their roles (e.g. hunter, teacher, and elder), and gender is included only when analytically meaningful or self-identified during the interview and consented to. This approach aims to balance clarity with respect for participants’ privacy and avoid unnecessary essentialism.

### Community workshop

**Fig. 4:**
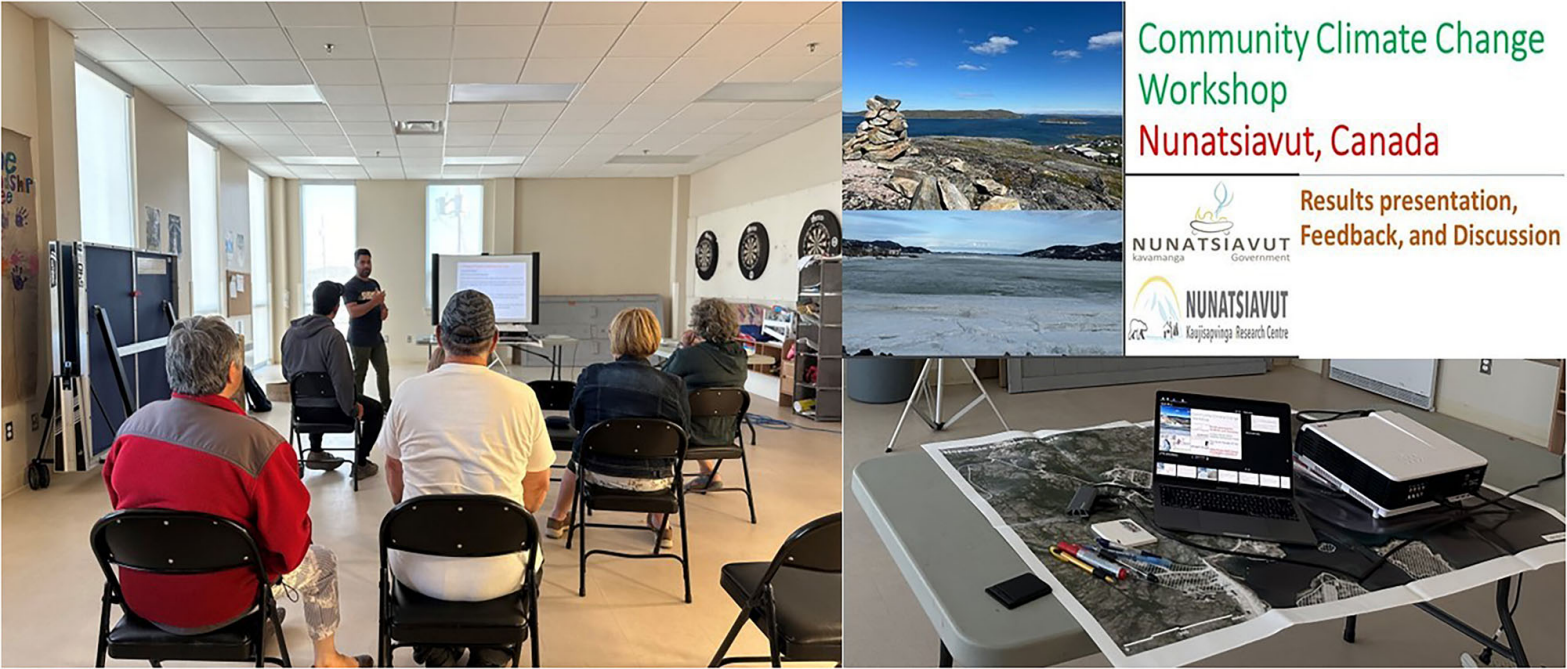
Community workshop involving presenting and validating results and gathering feedback from community members.

A workshop was conducted in Hopedale to present preliminary findings to the community (Fig. 4), validate the research results, and gather feedback. The workshop was designed to be an interactive session where community members discussed the findings and provided their input on how the research could better reflect their experiences and priorities.

#### Data analysis

##### Thematic analysis

All interviews were transcribed verbatim and analysed using thematic analysis. The themes identified during the community engagement served as the initial coding framework for the analysis, which was strengthened by additional themes that emerged inductively from the main fieldwork data (Table 3). The coding process involved systematically categorising the data according to these themes, followed by a detailed examination of how these themes interrelated. Coding was conducted manually by the lead researcher and reviewed in collaboration with community members and co-researchers to ensure consistency, accuracy, and cultural relevance.

**Table 3:** Research process.

Step 1	Engagement with NGRAC	• To ensure alignment of research goals with community priorities. • Incorporation of NGRAC feedback into research design.
Step 2	Community engagement	• To identify key themes and issues related to climate change adaptation. • Discussion with community members, key informants, and community leaders.
Step 3	Main fieldwork and data collection	• To collect in-depth data on the identified themes. • Interviews with five communities in Nunatsiavut: Nain, Hopedale, Makkovik, Postville, and Rigolet. • Semi-structured interviews, focus group discussions, key informant interviews, and interviews with community leaders.
Step 4	Community workshop and feedback	• To present findings to the communities. • Gather feedback.
Step 5	Data analysis	• Transcription and thematic analysis. • Feedback from workshop. • Coding. • Feedback from community members.

The data were analysed using a reflexive thematic analysis approach as outlined by principles of research ethics by the National Inuit Strategy on Research to ensure themes reflected Inuit worldviews, values, and lived experiences (ITK 2018). A combination of inductive and deductive coding was used: initial codes were guided by themes identified during early community engagement and co-researcher discussions (deductive), while additional themes emerged organically from the field data (inductive) during repeated readings of transcripts. Five thematic domains were developed: (1) lived impacts of climate change, (2) adaptation strategies and supports, (3) structural and economic barriers and limits, (4) colonial legacies and governance issues, and (5) cultural continuity and knowledge systems. This approach was chosen for its adaptability, emphasis on interpretation, and suitability for community-engaged and decolonial qualitative research.

##### Feedback

The workshop provided useful feedback and suggestions on the results presented, allowing for their refinement based on community responses, thereby enhancing the credibility, validation, and relevance of the research findings. It provided a deeper contextual understanding of adaptation and community concerns. Results were shared with community members in winter 2025, and additional feedback was sought to ensure the findings accurately reflected local experiences, concerns, and priorities. They were also shared through emails and Facebook messaging during 2024–2025. Knowledge translation efforts included plain-language summaries shared through oral presentations, emails, and Facebook messaging. These engagements ensured that results were accessible, relevant, and usable for local planning and advocacy. By contributing to local knowledge-sharing efforts, informing climate adaptation planning, and validating community-led observations, this study aims to provide direct and lasting benefits to the communities involved.

While this study offers in-depth insights into five Inuit communities in Nunatsiavut, the findings are context-specific and should not be interpreted as representative of all Inuit communities across the Arctic. However, the application of a political ecology lens and the findings could resonate with other Arctic regions and thus contribute to a growing body of climate research.

## Results

### Climate change impacts and adaptation

The communities in Nunatsiavut are facing significant challenges due to climate change and socio-economic pressures. Communities’ experience showcases that climate change is affecting hunting, gathering, travelling, economic stability, food security, traditional activities and knowledge of Inuit, and well-being (Table 4). It has a significant impact on traditional lifestyles, resulting in shorter hunting seasons, higher costs of living, and adaptation challenges. Across all five communities, members reported that they are experiencing later freeze-up and earlier thawing of sea ice, disrupting traditional hunting routes, food access, and wood gathering. Warmer temperatures have resulted in reduced ice formation and thinner, less reliable ice, posing safety risks, restricting travel, and causing stress, affecting mental health. Earlier springs, less snow, and drier summers affect access to traditional lands. Communities reported that these changes have disrupted the availability of traditional foods, with birds like geese and fish becoming smaller and less accessible and certain berries, such as blackberries, becoming less plentiful. This has increased the dependence on store-bought foods and technology. Some of these changes align with findings on ecological and ethnobotany studies in Nunatsiavut, including Inuit Knowledge studies by Nunatsiavut Government (2024), Norton et al. (2021), Rapinski et al. (2018), and Wolf et al. (2013).

**Table 4:** Key quotes illustrating impacts and adaptations in Nunatsiavut communities.

Impact	Adaptation
“In the last couple of years, sea ice has become thinner and softer, and is freezing later, which is bad for seals because seals are looking for places to get up on the ice. And the sea ice is melting faster, which is bad for us travelling to get seals, to gather wood for our homes, to get fish, birds, and everything. It’s affecting us in every sense”—Male elder, Nain.	“I have been recording weather conditions using my own weather station since 1992, when I was 23 years old. This is the first journal entry on 9 March 1992. I have recorded temperature −24 °C; sky conditions are clear; wind is LSW (Light South West); time of day is 6:10 a.m. On 19 April 1992: Easter Sunday, the temperature is −7 °C; sky conditions are snowing; wind is MNW (Moderate Northerly Wind); time of day is 7:45 a.m. On 19 April 2003: temperature is −8 °C; sky conditions are clear; wind is LSW (Light South West); time of day is 7 a.m. On 19 April 2024, the temperature is −2 °C; sky conditions are sunny; calm winds; time of day is 7 a.m.”—Male teacher, Hopedale.
“We don’t get a whole lot of snow, and I find, especially this year now with climate change, I think the weather is a lot warmer. Like we had an early spring. That doesn’t normally happen that early. It’s totally different”—Male, Postville.	“We are documenting and testing the sea ice before we travel on it, me and my son especially and some other people in town, documenting how solid it is, how thick it gets, and how far it goes. You can’t go if there are holes in the ice”—Male hunter, Hopedale.
“Berries are less plentiful; they dry up due to hot weather. Blackberries are not so plentiful because years ago you could go anywhere and pick them and you would have enough, but these days they are very hard to find”—Female food gatherer, Makkovik.	“Sometimes we get a logbook at our cabin. We write down when we test ice, where we test it, how thick the ice is, how the tides are, how the winds are, the amount of snowfall, put pictures on Facebook, and make it more aware for the younger generation that you should always be safe and test it instead of taking chances”—Inuit youth (male), Rigolet.
“We have lost a significant amount of time on the ice. A few years ago, sea ice would freeze up in late October or November and last until June or July. Now our ice only really forms in late December and is gone by either April or May. So that is about two to three months of ice loss”—Female elder, Rigolet.	“I am subscribed to the SIKU Ice Map. I turn that on when I leave town, turn the map on, and do the tracking. And when I go on the ice to test it, we take pictures, measure the ice, and send it to the SIKU site or put it on Facebook for people to see or share by word of mouth. That certainly changed from 30 years ago because the only way we would do it back then was by word of mouth”—Inuit youth (female), Nain.
“The main thing climate change affects is harvesting wood. The sea ice is freezing later in the fall. So, we have to haul more wood now during the spring if we get the chance”—Male hunter, Hopedale.	“People are buying more technology, diversifying the food sources. Technology is affecting big time and plays a big role. People are adapting their travel routes and gathering more wood in a shorter period”—Female hunter, Postville.
“On the land, it is soothing for the soul and mind, and when you can’t get out, it puts stress on people. When folks can’t access travel routes to go hunting and fishing, that has a mental strain. It is also a means of spiritual connection and provides mental stability”—Female key informant, Nain.	“People use data from weather stations and Environment and Climate Change Canada, and are using weather forecasting and satellite imagery a lot more to know if the ice is safe. So, it is no longer just depending on traditional knowledge only”—Female key informant, Makkovik.
“The traditional knowledge that has been passed down is changing. People’s travel routes are changing as the ice isn’t forming there or it’s breaking up faster”—Female, Hopedale.	“We make different routes to go to the places we want to go to hunt, fish, and gather wood. Now more and more people are starting to grow their own vegetables like potatoes, carrots, peas, radish, onions and turnips, and chicken and eggs”—Male hunter, Hopedale.

In response to challenges caused by climate change, communities have developed various adaptation strategies through a combination of technologies, traditional knowledge, and collective practices. As ice conditions become more unpredictable, some participants across communities noted that they document these changes through weather journals and logbooks. Community-based monitoring initiatives track environmental changes, wildlife populations, and vegetation, documenting their effects on ecosystems and livelihoods. A key activity is recording ice conditions and thickness. In Hopedale, some community members drill holes in the ice, using conductivity, temperature, and depth (CTD) Sensors to measure sea ice thickness, snow cover, depths, and water turbidity. This data helps understand freshwater distribution, its impact on river systems, and sea ice formation. Communities share information about ice conditions, trail access, berries, weather, harvesting, and daily activities on social media platforms such as Facebook.

Communities use technology for adaptation, especially for navigation and safety, utilising satellite phones, Handheld GPS, satellite messaging devices, boats, and modern snowmobiles designed for harsh Arctic conditions. Some community members now use iPhones for satellite messaging, enhancing connectivity in areas without cell signals. Wi-Fi devices are also used when going to cabins or harvesting to post updates on ice conditions and stay connected with family members. These technological advancements improve mobility and resilience in a changing environment. Communities use SmartICE’s SIKU maps for ice travel, weather forecasting for safe and efficient hunting routes, and websites like NASA Worldview (https://worldview.earthdata.nasa.gov/) to ensure safer travel. Technology, such as weather stations and satellite imagery, is used to supplement traditional knowledge and community resilience for better decision-making and adaptation. Hunters are adjusting by using longer, more inland routes to reach cabins and hunting grounds, though this increases gas consumption.

Traditional ecological knowledge plays an important role in adapting to changes in wildlife migration, ice safety, seasonal timing, weather conditions, and traditional foods. This knowledge, passed down through generations, is vital for survival and sustainability in the Arctic. It is taught to younger generations through classrooms and experiential learning on land. Inuit resilience, characterised by historical adaptability, plays a significant role in adaptation, ensuring the continuity of social fabric, cultural practices, and community well-being. Communities adapt to earlier ice thawing by harvesting wood earlier and stocking it for year-round use. The *“Buddy System”* helps manage rising gas costs by sharing fuel and machinery expenses for travel and hunting.

Despite a shift towards store-bought foods due to harvesting difficulties, community discussions, workshops, and information sessions on climate change provide comfort and solidarity. Community freezers and food-sharing initiatives by the Nunatsiavut Government and Inuit Community Governments across the five communities support food access and sharing. Such support systems have become crucial as traditional reliance on the land becomes more precarious.

Agriculture is emerging as an important adaptation strategy across the five communities, with growing interest in vegetable gardening. Communities cultivate various crops such as turnips, potatoes, onions, peas, tomatoes, radishes, berries, lettuce, broccoli, carrots, rhubarb, and strawberries. Natural fertilisers such as capelin, seaweed, and kelp are washed and dried before application. Vegetables are grown on small land patches, in constructed boxes, containers, and greenhouses. Some community members engage in poultry farming, raising chickens, and selling eggs.

Despite these adaptations, shorter winters and changing ice conditions continue to affect livelihoods, particularly in activities like wood gathering and hunting. The cumulative impact of these changes reveals a deep interconnectedness of climate, environment, economics, and well-being in communities.

### Historical and contemporary construction of risk

The legacies of colonialism, post-colonialism, and capitalism, which collectively amplify the impacts of climate change, escalate living costs, and create adaptation challenges, are deeply intertwined with the historical and contemporary construction of risk in Nunatsiavut Inuit communities. Colonial and post-colonial policies disrupted traditional Inuit ways of life and knowledge, forcibly relocating communities, forcing sedentarisation, undermining traditional economies, and imposing a market-based system (Kennedy 1982; 2015; Ben-Dor 1966; Brice-Bennett et al. 1977; 2023) that prioritised colonial and later capitalist interests over Indigenous sovereignty and sustainable living practices. This disruption not only weakened Inuit self-sufficiency but also made communities increasingly dependent on imported goods and technologies, driving up the cost of living and making communities more dependent on global market fluctuations. The legacy of colonialism is evident in the persistent economic inequalities and social vulnerabilities that continue to affect Inuit in Nunatsiavut, where the impacts of climate change intensify their exposure to risk.

Climate change exacerbates these vulnerabilities by altering hunting patterns, reducing sea ice, increasing the cost of living, particularly for food and energy, and affecting the availability of traditional food sources, thereby increasing the need for costly, store-bought food as experienced by community members. The rising food prices and technological dependencies compound these challenges, making it harder for Inuit communities to maintain their cultural practices and economic self-sufficiency while also increasing their exposure to environmental and economic uncertainties. A key informant in Hopedale narrates, *“[the] Corporate world has pushed us away from our traditional ways of living and increased our dependence on costly store foods. Its aim is economic exploitation.”* A community member in Nain mentioned, *“The market is forcing us to change our traditional subsistence ways of life and adopt new greedy ways, which detach us from our land and culture of going out hunting and gathering.”*

An Inuit youth in Hopedale, while explaining the nexus of capitalism and rising living costs, expressed, *“Inuit and all other Indigenous Peoples and people living in remote communities in the Arctic are affected by the cost of living directly from the higher level of corporations. It is not our fault that we have to pay more; it just happens because of those powers above us in the economy. So, I think the ones who are in control and the government are responsible for these problems.”* Another participant in Hopedale mentioned, *“In Inuttitut, I automatically think about the word inogatta meaning because we are Inuit or inoKatigegatta meaning because we are fellow Inuit, and there is no difference between us and people in other parts of the world. When it comes to Inuit knowledge, everybody knows and respects the land as number one. Respecting nature, respecting the wildlife. We don’t want to trample and take down wildlife because of greed. The land in itself is living, and it has so much more that benefits our survival when we work with it. Now big private companies ruin our land and lead to deforestation and pollution, say, to set up a big corporation that leads to people making big dollar profit and being greedy.”*

The dependence on technology and modern infrastructure, which often faces adaptation challenges in the Arctic environment, introduces additional economic burdens and risks, as the high costs associated with maintenance and operation are unsustainable for many Inuit families. Governance frameworks and provincial and federal control over resources also played a crucial role in shaping the current challenges faced by Nunatsiavut Inuit, as these policies have marginalised Inuit voices, limited their participation in decision-making processes, and failed to recognise their rights to self-determination and self-governance. A community member in Rigolet explained, *“The big businesses and federal and provincial governments try to make a profit off our land and isolate us from it. They look at the land as nothing and as just a heap where they can have it as their playground. But to us, it is our everything. It is our home. It is where we thrive and is a huge part of our well-being.”*

The forced relocation of Inuit in Nunatsiavut from northern Labrador in Hebron and Nutak is an important factor in the historical construction of risk. Several community members in Nain, Hopedale, and Makkovik reported that in 1956, the community of Nutak was forcibly relocated from the northernmost part of Labrador to other Nunatsiavut and Labrador communities, and then three years later, in 1959, the community of Hebron was relocated. These relocations brought almost 500 Inuit from northern Labrador, considered the historical stronghold of independent Inuit, to the south (Brice-Bennett et al. 2023; Evans 2012).

This introduced a range of challenges for those relocated to communities that were very different lifestyle-wise. There was a lot of tension in some of the communities. An elder in Nain, while recounting the memories of the relocation, narrated, *“It was a very heartless relocation; it was horrible and the root cause of a lot of issues that persisted for a long time afterwards. Taking all these people and moving them far away, somewhere from their context, was hard.”* An elder in Hopedale narrated, *“Around 200 people came from Hebron. I came here in 1959. We stayed in tents; nobody helped.”* A key informant in Makkovik said, *“The relocations have happened elsewhere in Inuit Nunangat, but this area was pretty recent in the 1950s. About 200 years ago, around 800–900 Inuit were living in the northern part of Labrador, and now there is none living in the north of Nain.”* Now Nain is the northernmost area where people live. Community members in Nain recounted that many people who were relocated from Okak and Hebron were relocated to as far south as Makkovik or Upper Lake Melville (in central Labrador), where a large population of Inuit live.

The relocatees mixed with communities that often included settler and Kallunangajuit populations rather than the majority Inuit-speaking Inuit populations in far northern Labrador. Participants across the communities noted that many people living in Nunatsiavut’s five communities are the descendants of people or have grandparents who were very young when they were forcibly moved or grew up seeing the challenges relocatees faced relative to others. The communities also have a close connection with the Torngat Mountains National Park because a lot of them are descendants of people who would have lived in that area. A community member in Nain mentioned, *“Torngat Park does not have anybody living there, but that does not mean there is not a rich cultural history there.”* Another community member in Makkovik said, *“There is more linkage between Inuit history and culture to Torngat Park than there is to other areas around where some of the communities are today because some of the communities are recent, and experience to Torngat Park was hundreds of years until the 1950s.”*

The communities that were forcibly displaced had to travel a lot of distance to the south. An elder in Nain narrated, *“The government showed up and told us that we had to leave. We were being forced to leave because the government said they were not going to supply any additional services or help us if we have trouble or anything. So, we had to move quickly.”* Relocatees recollected it as one of the saddest aspects of Labrador history. In the years after, there were lots of tensions and challenges. The relocatees faced a lot of difficulties in getting food; as a community member in Nain mentioned, *“Because you get moved from your hunting grounds you knew very well to a new place where others have their hunting grounds established, and you do not know the area.”* There was a tremendous amount of racism towards those coming from the north because they looked and lived differently than the ones in the communities that they moved into; as a community member in Makkovik mentioned, *“it was a real challenge; the new place brought its own complications. It was not like home.”* An elder participant in Hopedale said, *“The forced relocation felt like our identity and land were taken from us, and it affected how we could hunt and prepare for the future.”* Community members in Nain, Hopedale, or Makkovik reported that most of the descendants of the relocatees ended up, over the long term, either in Nain, Hopedale, or Makkovik. Nain and Hopedale are the northernmost communities and have the largest number of people who are descendants of those living in the north.

External agencies, driven by colonial and commercial interests, have attempted to influence Nunatsiavut Inuit social transformation by altering their harvesting practices. Participants across the communities recalled that the missionaries criticised Inuit sharing customs as incompatible with settled life and Moravian goals of “civilising” Inuit through the doctrines of Protestantism and capitalist ethics, advocating for “rational” economic behaviour aimed at individual accumulation of property (Kennedy 1977; Kleivan 1966). Capitalism, modernisation, economic development ambitions, and cultural essentialism seek to influence land claims agreements and policies, resulting in policies that discourage Inuit harvesting. These include restrictions on harvesting and wildlife product disposition, preferential treatment for sports harvesters, habitat destruction from industrial development, and exclusion of Inuit from economic strategies and policy making (Natcher et al. 2012; 2020; Snook et al. 2022). By limiting Inuit control to subsistence activities, these efforts enabled governments to dominate and profit from commercial fish and wildlife industries, reflecting a form of neocolonialism and capitalist interests where economic and political power is exerted to marginalise Inuit economic autonomy and sovereignty and maintain control over Indigenous resources.

### Barriers and limits to adaptation

#### Environmental injustice, structural inequities, and economic constraints

Nunatsiavut communities are small, ranging from approximately 180 residents in Postville to 1200 in Nain, and are disproportionately impacted by climate change. These impacts constrain communities’ capacity to adapt and create environmental injustices, as Inuit—whose livelihoods and well-being are closely tied to the land and natural resources—face inequitable access to the means of adaptation. Climate change exacerbates existing social, economic, and environmental vulnerabilities, deepening systemic inequities.

Access to food sources is increasingly challenging due to changes in climatic and sea ice conditions. Several community members do not have the necessary means and technology, like skidoos, trucks, boats, GPS, and satellite phones, to travel, hunt, and gather food to sustain themselves. Climate change complicates these issues by exacerbating the challenges of accessing land, sea ice, and food sources, creating social and adaptation inequalities. Community members across all five communities, reported that the price of skidoos is expensive, and they are more electronic, so it is harder to work on them if they break down. This is further complicated by the availability of insufficient snowmobile repair services. Participants noted that some of the local mechanics have the necessary skills and training to repair skidoos, boats, and trucks, but a lot of times they are transported to Happy Valley-Goose Bay to get them repaired, which is expensive and time-consuming, affecting hunting, food gathering, and travelling.

Participants across communities narrated that some elders are not able to hunt, travel, and collect firewood and find it particularly hard to adapt. They depend on community food and firewood sharing networks and community freezers. Participants mentioned that adapting to the cost of living is exacerbated by climate change, which affects gathering traditional food and intercommunity travel. The high costs associated with necessary tools—such as skidoos, boats, and advanced hunting equipment—place a disproportionate burden on economically disadvantaged households. This disparity is a classic issue in political ecology, where access to resources is unevenly distributed, often leaving marginalised groups more vulnerable to environmental changes.

The economic constraints in Inuit communities of Nunatsiavut are deeply rooted in historical and structural inequalities. The challenges, such as the high cost of living and adaptation, stem from broader systemic issues, including colonial histories and capitalism, that have marginalised Indigenous Peoples and risked their economic activities. The reliance on expensive store-bought foods signifies a loss of traditional subsistence practices, driven by climate change, affecting ways of life.

Inuit in Nunatsiavut are using skidoos, all-terrain vehicles (ATVs), boats, and trucks to travel for hunting, food gathering, intra- and intercommunity travel, and cultural activities. The reliance on technologies such as snowmobiles, trucks, and boats for transportation, GPS systems for navigation, satellite phones for communication, and advanced fishing equipment has grown as traditional methods become less viable due to climate change. The increasing use of modern technologies also reflects broader trends in modernisation, highlighting the intertwined nature of modernisation and climate change. However, these technologies are costly to purchase and maintain, placing a significant financial burden on community members. Wealthier households adapt better to climate change due to their financial resources, while lower-income families, reliant on traditional practices, face greater vulnerability.

Rising costs of fuel and store-bought food, combined with inflation, exacerbate economic strains, forcing many community members to rely on communal resources such as community freezers and community sharing networks. An Inuit youth in Postville mentioned, *“It is getting difficult to get some of the traditional foods. So, we rely more on the store-bought food, but at the same time it is getting expensive. And even going out on the land, buying gas and equipment, is also expensive. When it comes to the rising cost of living, there is no win to it. It’s like there is nothing you can do; it just keeps on rising.”* Communities’ efforts to use agriculture for growing their own vegetables as a means to adapt to rising store-bought food prices are hindered by the lack of availability of soil (e.g. in Hopedale). Consequently, community members collect soil from the surrounding lands and transport it home to cultivate vegetables, but it is insufficient to support agriculture on a large scale.

The economic strain extends to the high cost of store-bought food, which has become a necessity due to reduced access to traditional hunting areas and less availability of traditional foods like caribou, geese, seals, partridges, and berries. Adapting to climate change in Nunatsiavut comes at a high economic cost, particularly for communities increasingly dependent on expensive technologies for survival. A community member in Rigolet narrated, *“Some people have to choose between eating or paying their bills for heat because everything has changed and the cost has gone up, and if you cannot get off on the land to get your traditional country food, then you have to rely on stores, but people cannot always afford to buy from the store.”* Another member in Hopedale narrated, *“Buying new technologies is not affordable for everyone. It is very expensive to buy a new skidoo now. And sometimes it is not always worth the value of the money that you pay for because the season has gotten so short that we do not get to use our snowmobile for as long as we did years ago.”* A community member in Postville explained how the cost of basic food items is getting expensive to buy and said, *“The prices are rising every year on store-bought foods, especially for the basics like milk, sugar, flour, frozen goods, and meat, which people depend on to cook and get by with. Everything is going up. All the prices are going up. Gas, food, electricity.”*

The financial strain limits the ability to invest in both immediate adaptive measures—such as maintaining equipment, adjusting travel routes, harvesting firewood earlier in the season, or sharing fuel—and longer-term strategies like purchasing efficient machines, or supporting community monitoring initiatives, thereby perpetuating cycles of vulnerability and dependence. New machines such as skidoos and boats are essential for accessing hunting grounds due to their improved reliability and fuel efficiency over older models; however, their high purchase cost poses a significant barrier for many community members. One significant disadvantage is the increased difficulty in repairing these machines when out on the land. This concern is largely rooted in the historical unreliability of snowmobiles, which often required frequent repairs in remote locations. The economic burden forces many members across the five communities to limit their travel, share resources, and contribute to the community freezers, reducing their overall ability to adapt to changing conditions. It affects the cultural core of the communities, as a member in Makkovik explained, *“An important part of our culture is providing food for people, especially those who do not have as much access, like elders and low-income families, and sharing wild food like seal meat. So, that definitely impacts the culture, health, and well-being of the community if hunters are not able to get out on the land during certain times of the year that we used to do.”*

A participant in Hopedale narrated, *“So many people just go to the store, and they pay money and pick up the food and eat it. It is so different than when you go hunting; you put so much time and effort and strength in your mind and body to harvest food. You really appreciate it when you eat food that you harvested or food that was shared with you. In our culture, we call that Pajuk (pronounced as Payuk). So, when you share your food and you love it, and learn how to cook it. It’s really good. That’s living. Now climate change is affecting it, and it is hard to adapt to these new ways of life.”*

#### Expensive energy and fuel prices, and economic inequality

Communities use wood, electricity, furnace heat, and stoves for heating and lighting their homes. They rely on diesel generators for electricity, but fluctuating fuel prices and extreme weather conditions make it challenging to maintain stable and affordable energy supplies. Participants across communities noted that while many households continue to use traditional woodstoves for heating, some have adopted modern, energy-efficient stoves through the Nunatsiavut High Efficiency Woodstove Programme, which consume less wood and retain heat for longer periods. Some families have installed heat pumps through the Nunatsiavut Energy Efficiency Retrofit (NEER) programme, depending on financial eligibility. A participant in Hopedale mentioned, “we had to pay two thousand dollars out of ten thousand dollars for our heat pump.” Access to both programmes is income-dependent, with lower-income households eligible for full subsidies, while others, depending on their income level, are required to cover a portion of the total cost. However, these stoves are highly expensive and unaffordable for most families. Many community members have switched to electric heating, but the high cost of electricity complicates this option.

The price of fuel is high in Nunatsiavut. A participant in Nain said, *“The gas is expensive, which affects hunting, travelling, and cultural activities. We might have the most expensive gas in the country. It is more expensive here than in cities like Quebec or Ontario. It is even more expensive here than further north.”* Across the five communities, members use skidoos and boats for hunting and food gathering, but some cannot afford the gas prices and adapt by sharing costs and machinery with others, known as the *buddy system* or *gas buddies*.

Due to changing sea ice conditions, community members described needing to take longer routes for hunting and gathering, which increases gas consumption. A community member in Hopedale explained, *“We are taking different routes to go to our cabins and for hunting. My cabin is 38* *km by boat and probably 34* *km by Skidoo, and to get to our cabin by boat is probably 40–50* *min with the regular straight route and 30* *min on Skidoo. And with this new route, the last couple of years we have been taking, it takes me two to 3* *h on Skidoo to get to the cabin as compared to 30* *min. It is anywhere from two to four times as far, with more gas and more inland routes.”* Another member in Hopedale said, *“I got a 900 ACE, which is a four-stroke. One way to the cabin, I probably burn a gallon and a half, maybe. By boat, depending on your load, but average of four to six gallons. And now, with the different route for Skidoo, we are burning the same amount as a boat, or more, because we have got to go farther inland to get to the cabin. Before the ice is solid enough to drive on a straight route, it is taking two to three times as much gas on Skidoo as compared to 15 years ago.”*

Community members face uncertainty in obtaining traditional foods, sometimes returning empty-handed despite using large quantities of fuel. Caribou (George River Caribou) has been an important part of the diet and culture of Inuit in Nunatsiavut, but due to a drastic decline in their numbers, which communities attribute to a lack of food caused by climate change, their hunting was banned within community lands in 2013. To hunt caribou, some community members travel to Torngat Mountains National Park, but the travel costs from all five communities are high, discouraging many from hunting caribou. This affects food security and contributes to a reliance on store-bought foods.

Across the five communities, community members rely on sea ice to travel long distances for wood collection. Climate change, however, has disrupted this practice by causing the sea ice to freeze later and melt earlier, reducing the available time for gathering wood. As a result, some members have adapted by purchasing wood locally or from Happy Valley-Goose Bay. This adaptation, while necessary, is costly due to the high price of wood and the additional transportation expenses from Happy Valley-Goose Bay, and sometimes low-quality and expensive wood is sent to the community members. A community member in Hopedale mentioned, *“For here in Hopedale, especially, we don’t have much wood available. The other four communities got a lot of wood around their towns, but there are a lot of people that do not have snowmobiles. A lot of people do not have good-paying jobs to buy snowmobiles, to get gas, wood, or to heat their homes. So, they are relying on a few people that provide, say, Southern Labrador or Goose Bay, to buy wood and then to ship up on the ferry boats in the summer or in the fall.”* Another member in Hopedale mentioned, *“If people got money, they would buy wood from locals or from Goose Bay, which is too costly and time consuming.”* A key informant in Rigolet mentioned, *“With climate change, it is getting difficult to adapt, particularly for seniors on fixed incomes, due to the high expense of food, sustaining country foods, and also heat, because some homes depend on just wood heat, or wood heat is the preferred choice of heat.”*

The communities’ energy reliance causes environmental strain and economic disparities, exacerbating adaptation barriers. Economic disparities are evident as a large number of community members are unable to afford modern, energy-efficient stoves or the high cost of electricity and gas, forcing them to rely on less efficient options (Nunatsiavut Energy Security Plan 2016). This illustrates the complex interplay between environmental policies, economic inequalities, and cultural dynamics.

#### Infrastructural deficiencies and geographic isolation

Community members across all five communities reported that significant infrastructural deficiencies pose barriers to effective adaptation for Inuit in Nunatsiavut. The lack of road access and reliance on air and seasonal sea transport make it difficult for communities to receive food, goods, and services, particularly during extreme weather events or when ice conditions prevent travel. The stores usually run out of food during winter and spring. The absence of reliable infrastructure restricts access to essential supplies and hinders emergency response efforts. For example, many participants suggested the need for a road to Happy Valley-Goose Bay to reduce costs and improve access to goods and services. However, the construction of such a road is controversial due to potential environmental impacts, such as disrupting wildlife and their migration routes and greater access for people from other places to harvest fish and wild foods in Nunatsiavut. The lack of adequate healthcare facilities and the unavailability of doctors also significantly impact the well-being of the community. As a result, participants across communities reported that they are often compelled to travel to distant locations such as Happy Valley-Goose Bay, St. John’s, Labrador City, or other southern places for medical consultations. These journeys are not only expensive but also time-consuming, with community members sometimes spending nearly a week on round-trip travel for medical appointments. This situation imposes economic constraints on Inuit communities, exacerbating their financial challenges.

These infrastructural deficits are not merely logistical issues but are deeply rooted in historical marginalisation, power dynamics, historical governance decisions by provincial and federal governments, and systemic neglect by broader political and economic systems. The lack of adequate infrastructure, such as road networks and robust community facilities, can be traced back to policies that have historically prioritised urban and southern Canadian development at the expense of Indigenous communities in the Arctic (Royal Commission on Aboriginal Peoples 1996; Royal Commission on Labrador 1974; Hanrahan 2003; Bowers et al. 2023). This marginalisation is further compounded by the geographic isolation of Nunatsiavut, which is both a natural and socially constructed barrier. The absence of reliable air transportation networks and the dependence on seasonal sea routes for goods and services reflect a broader lack of investment in the region’s infrastructure. This geographic and infrastructural marginalisation limits immediate adaptive responses, restricting access to external resources, emergency services, and economic opportunities. It hampers initiatives such as the installation of energy-efficient heating systems, year-round food supply, and safe travel to hunting areas, thereby reinforcing the vulnerability of Nunatsiavut communities to climate impacts.

### Adaptation for whom?

“Adaptation for whom?” in the context of a political ecology of climate change adaptation underscores inequities in adaptive capacities among community members. The results highlight that adaptation efforts are not uniformly utilised across communities. Wealthier households and those with better access to resources are more capable of adopting new technologies and strategies to cope with climate change. In contrast, economically disadvantaged households, particularly those reliant on traditional livelihoods, face significant barriers due to the high costs of essential tools like skidoos, boats, GPS, and satellite phones, which are important for accessing traditional hunting grounds and food sources. According to participants, the economic burden is exacerbated by the high cost of store-bought food, which is often of lower quality and nutritionally inadequate, and has become a necessity due to reduced availability of traditional foods, access to traditional hunting areas, and transportation challenges. The rising prices are not limited to food; fuel prices are also a major concern, creating additional financial strain.

A political ecology lens reveals that economic burdens are not evenly distributed, leading to disparities in food security and overall resilience. Participants across communities noted that systemic issues—such as inadequate infrastructure and limited provincial and federal government support—continue to disproportionately affect Inuit in Nunatsiavut. This includes insufficient funding for climate-resilient infrastructure, slow responses to community-identified needs, and limited coordination with Inuit-led initiatives. The economic challenges within communities are interconnected, forcing difficult decisions between essential needs like heating homes and purchasing food. These complex and intertwined issues are further exacerbated by the impacts of climate change, intensifying the existing vulnerabilities and economic pressures.

A political ecology lens foregrounds the structural forces that shape who gets to adapt to climate change—and at what cost—shifting the focus from technical adaptation to the socio-political terrain in which it unfolds (Taylor 2014; Agathangelou and Killian 2022; Newell 2021; Kalt 2024). Far from being neutral or universally accessible, adaptation is deeply embedded in unequal relations of power, historical dispossession, and differential access to resources and knowledge (Robbins 2019; Nightingale 2016; Escobar 2021). The question “adaptation for whom?” thus lies at the core of political ecology, revealing how vulnerability is socially produced and how adaptive capacities are unevenly distributed across and within communities. It draws attention to how colonial legacies, economic marginalisation, and infrastructural exclusion shape differential capacities to adapt to climate change across and within Inuit communities in Nunatsiavut. This aligns with political ecology’s focus on how power, history, and uneven development condition access to adaptive resources and define who bears the burden of risk. As one participant in Nain noted, *“People often struggle very much. And oftentimes, folks have to make a decision of either warming their homes or getting food. And if they make a decision for one, they can’t do the other.”* This illustrates the compounding vulnerabilities where economic strain translates directly into constrained adaptive options.

In Nunatsiavut, political ecology framing reveals a complex interplay of colonialism, capitalism, and environmental marginalisation. While Inuit have long demonstrated resilience and adaptability, present-day responses are increasingly constrained by the affordability of equipment, geographic isolation, and reduced access to safe land and traditional practices—conditions shaped by political and economic histories, and climate change. In the context of this, a participant in Makkovik mentioned, *“If you’re trying to go out on the land to get food, you are spending more gas going the opposite way due to changing enviornmental conditions. It’s very expensive. A can of gas is almost $50, which is too expensive for some members.”*

Across the five communities, participants consistently described how the high cost of fuel, food, and hunting equipment creates significant disparities in who can engage in land-based practices. *“Some people cannot afford the price of gas or food. Now even the price of hunting equipment has increased, which is difficult for some to bear. For some, there is always a dilemma about what to prioritise”*, one participant in Rigolet noted, underlining how economic precarity limits even basic access to the land. Another participant in Postville echoed this: *“It’s not affordable to everyone. The price of skidoos, and boats… it’s difficult for folks to have the things they need to go out hunting”*. In this way, adaptation is constrained not by individual will but by structural forces rooted in colonialism, capitalism, and infrastructural marginalisation.

Political ecology foregrounds the importance of historical processes in shaping present-day vulnerabilities (De la Hoz et al. 2024; Malik and Ford 2025a; Fernando 2020). Participants across the communities spoke of the psychological and cultural impacts caused by rapid environmental changes, recalling how predictable seasons enabled secure harvesting patterns. A participant in Hopedale noted, *“for many generations, you harvest certain animals during certain times of the year, and you work in harmony with nature. But if climate change continues then it’s going to make it harder to adapt.”* These testimonies reflect what Nixon (2011) terms *slow violence*—gradual environmental degradation that accumulates over time and disproportionately affects those already structurally marginalised.

Internal colonialism resonates with the experience in Nunatsiavut. Participants across the communities voiced concerns over the high cost of fuel, significant increase in airfares, and inadequate infrastructure despite proximity to supply routes. A participant in Rigolet noted, *“we are paying more for a litre of gas than the southern communities. But yet they [oil tankers] stop here first. That doesn’t make sense”*. This reflects a wider critique of state disinvestment in remote Indigenous communities and the prioritisation of industrial over community needs, underscoring how climate risk is both materially and politically produced. These struggles are compounded by infrastructural and logistical marginalisation. With no road access, communities depend on air or seasonal marine transport, which limits both the timely provision of goods and the responsiveness to climate events. This geographic isolation, a legacy of both settler colonial policies and underinvestment, hampers community resilience-building. *“We don’t have roads anywhere. It’s either fly-in or ship-in, and that adds to delays in food and basic supplies,”* said one participant in Makkovik.

**Fig. 5:**
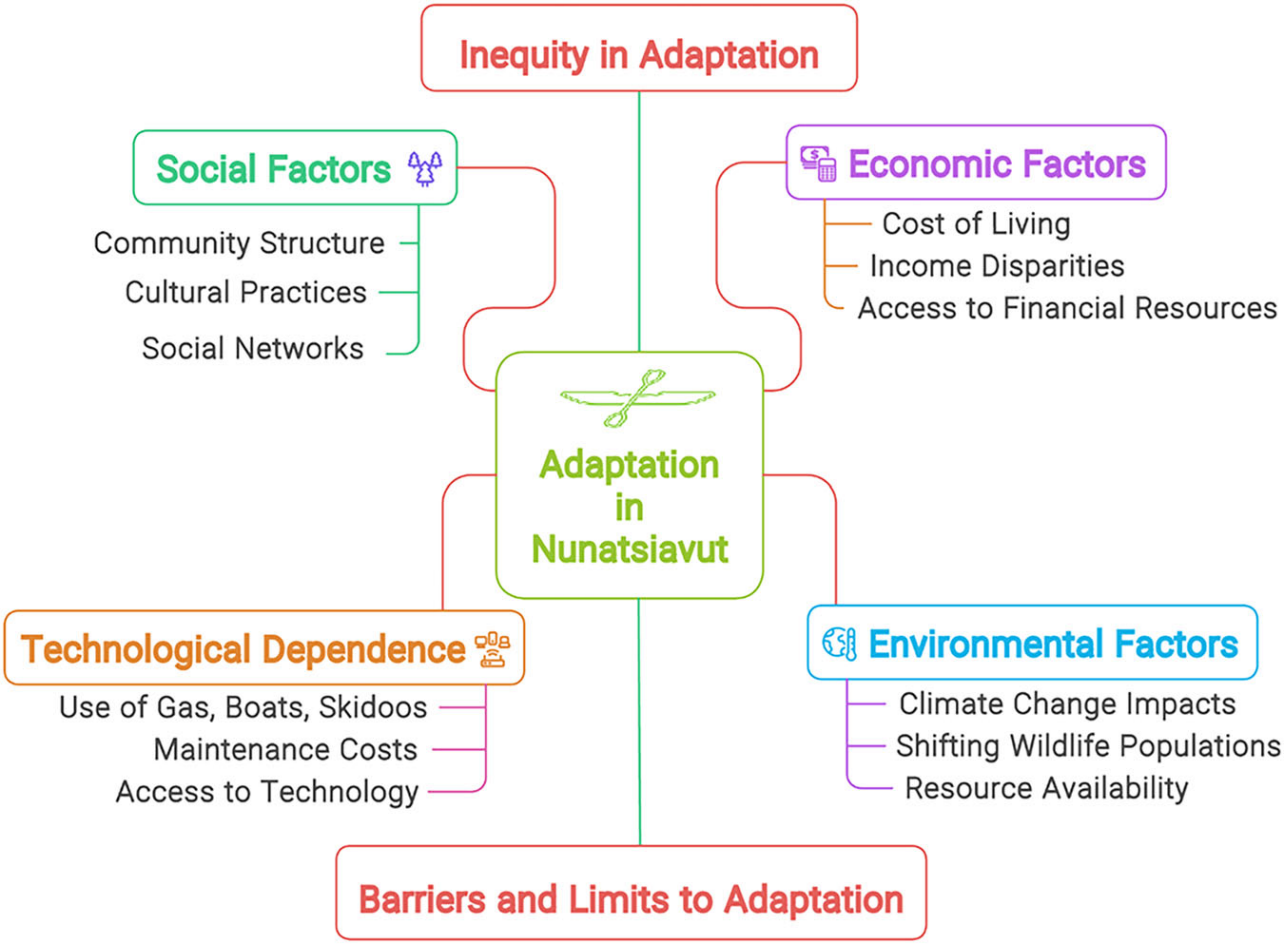
Political ecology of climate change adaptation in Nunatsiavut, illustrating the interconnected social, economic, environmental, and technological factors influencing adaptation. The diagram highlights the central question of ‘adaptation for whom?’ and emphasises the inequities and barriers present in the adaptation process.

Figure 5 presents a visual synthesis of the political ecology of climate change adaptation in Nunatsiavut, illustrating how social, economic, environmental, and technological factors intersect to shape adaptive capacity. At the centre is the critical question “adaptation for whom?”, which highlights inequities rooted in colonial histories, socio-economic marginalisation, and uneven access to resources. Social factors such as community structure, cultural practices, and kinship-based sharing networks support adaptation but are under pressure due to cultural erosion and changing land use. Economic constraints—including high living costs, income disparities, and limited financial access—limit the ability to acquire necessary tools for land-based activities. Technological dependence, such as reliance on skidoos, boats, and GPS, aids safety but introduces cost barriers and maintenance burdens. Environmental changes, including unpredictable weather, shifting wildlife, and reduced resource availability, directly impact food security and traditional practices. The figure underscores that adaptation is not simply a response to environmental shifts, but a process shaped by complex systems of power, access, and historical injustice.

The question of “adaptation for whom?” thus becomes important in understanding the uneven distribution of both resources and risks. Addressing these inequities requires targeted policies that consider the unique challenges (and opportunities) faced by different groups, ensuring that adaptation efforts are inclusive and equitable. This approach must involve strengthening local institutions, enhancing access to financial resources, and supporting traditional practices that are culturally relevant and sustainable. By focusing on these aspects, this research calls for a more just and resilient approach to climate change adaptation in the Arctic.

Analysing the barriers to climate change adaptation in Nunatsiavut through a political ecology lens reveals that these challenges are deeply embedded in historical, socio-economic, and political contexts. Effective adaptation strategies must therefore go beyond technical solutions to address the systemic inequalities and power dynamics that shape vulnerability and resilience. This includes advocating for equitable resource distribution, infrastructural investment, and the integration of traditional and diverse knowledge systems.

## Discussion

Little attention has been given to the political ecology of climate change in the North American Arctic, with most work in this area focusing on the European Arctic (Benjaminsen et al. 2015; Cavanagh and Benjaminsen 2017; Benjaminsen and Robbins 2015; Benjaminsen 2015). This study, to the best of our knowledge, is the first to examine the political ecology of climate change adaptation in Nunatsiavut.

Many Inuit in Nunatsiavut face significant food insecurity, economic inequality, and poverty, rooted in both historical and contemporary impacts of colonialism, capitalism, and structural inequity (Nunatsiavut Government 2024; Dwyer-Samuel 2024; Bowers et al. 2023; Kourantidou et al. 2021; Brice-Bennet 1977). Settler colonialism and internal colonialism are important for understanding Indigenous-settler relations in Canada, where Indigenous Peoples experience dispossession and marginalisation. Scholars have drawn on both settler colonialism and internal colonialism to theorise the ongoing subjugation of Indigenous Peoples. Settler colonialism refers to a structure that aims to eliminate Indigenous presence in order to secure land and resources for settlers (Liboiron 2021; Perry 2023). In contrast, internal colonialism emphasises the ways in which Indigenous communities are politically and economically marginalised within the borders of the settler nation-state, often treated as internally colonised subjects akin to the Global South (Das 2020; Bernauer 2024). In the context of Nunatsiavut, both frameworks are relevant: settler colonialism underpins the historic dispossession and forced relocations experienced by Inuit communities, while internal colonialism is reflected in the systemic neglect, limited infrastructure, and jurisdictional fragmentation that continue to constrain Inuit self-determination and climate adaptation today. Inuit in the Canadian Arctic have been subjected to colonial dispossession, injustices, and subjugation (Bernauer 2024) and are experiencing ongoing elimination and dispossession through various neocolonial and capitalist practices.

Various interconnected factors like climate change, poverty, high living costs, high cost of machinery, inadequate infrastructure, intergenerational trauma, and forced relocations have affected and are continuing to affect Inuit in the Arctic (Dombrowski et al. 2016; Ayeb-Karlsson et al. 2024; Natcher et al. 2012; ITK, 2021; Cunsolo and Ellis 2018; Middleton et al. 2020). The median after-tax income for Inuit in Nunatsiavut is $31,400 compared with $49,600 for non-Indigenous people living in the region and $37,200 for non-Indigenous Canadians as a whole (Statistics Canada 2022). Newfoundland and Labrador province has the highest rate of unemployment in Canada at 10.1% (Food Banks Canada 2024).

In Nunatsiavut, those most affected include low-income families and elders. Colonial policies, such as relocations, have historically restricted Inuit mobility and participation in traditional harvesting activities, limiting the transfer of essential life skills and self-determination in food systems. For Hebronimiut, the announcement to relocate took place in a church where they could not protest or speak due to the belief that the church should not host controversial matters, and they were also not provided an interpreter (Brice-Bennett 2017). They voiced their concerns in a letter written in Inuktitut, translated by Rita Andersen, as “We are asking not to be removed from our community because we are used to our traditional ways of hunting and it is very excellent. Hunting seals, char, caribou and other animals is our livelihood and are plentiful” (Brice-Bennett 2017, p. 98). The *Avanimiut* (meaning “people of the North” in Inuktitut) maintained their traditional ways of life and beliefs, resisting pressures from European colonisers, including Moravian missionaries and the Hudson’s Bay Company, and the long-term impacts of colonialism (Brice-Bennett et al. 1977; 2023).

Inuit in Nunatsiavut and other places in the Arctic are experiencing loss of biodiversity, language, culture, and transfer of traditional knowledge due to climate change (Cuerrier et al. 2019; Kourantidou et al. 2021; Pearce et al. 2011; Naylor et al. 2023). Caribou population is declining in Nunatsiavut and across the Canadian Arctic (Johnson et al. 2024), impacting well-being and resulting in the loss of cultural knowledge (Schmelzer et al. 2020; Borish et al. 2021). The ban on caribou hunting in Nunatsiavut has affected Inuit cultural continuity, identity, mental health, food security, and adaptive capacity (Borish et al. 2021; Cunsolo et al. 2020). Later freeze-up and early melting of sea ice changes are affecting travel, food, and wood gathering (Ford et al. 2023; Cooley et al. 2020; Wilson et al. 2021).

This study highlights the barriers and limits to adaptation in the context of rapid environmental changes and socio-economic constraints. In some cases, the barriers to adaptation are so significant that they push the limits of what is feasible, raising concerns about the long-term resilience of the community. The study emphasises the need for environmental justice in adaptation strategies, which aligns with political ecology’s focus on addressing power imbalances and social inequalities. Ensuring community participation and equitable access to resources is important for effective and just adaptation, addressing the interconnected social, economic, and environmental challenges faced by Inuit in the Arctic.

This study demonstrates how a political ecology framework can critically illuminate the uneven terrain of climate change adaptation in the Arctic. Rather than viewing adaptation as a set of technical solutions or behavioural shifts, political ecology contends that colonial legacies shape climate futures and differential capacities to adapt (Simon and Kay 2024; Nightingale 2016; Robinson et al. 2023). By situating adaptation within systems of power and access, this approach makes visible the structural roots of vulnerability and the socio-political conditions that constrain local agency (Eriksen et al, 2015; Vigil 2024; Taylor and Bhasme 2021).

While Inuit communities in the Arctic continue to demonstrate resilience and adaptation through monitoring and forecasting products such as Windy.com, tide tables, weather and marine forecasts, and SmartICE (Simonee et al. 2021), the ability to adapt is profoundly shaped by factors beyond individual or community control (Malik and Ford 2025b; York et al. 2024; Vogel and Bullock 2021). High costs of fuel and equipment, dependence on ageing infrastructure, and limited institutional support present significant barriers—especially for lower-income households (Ahmed 2025; Strand 2018; Povoroznyuk et al. 2022). These conditions are not incidental but reflect deeper patterns of uneven development, commodity extraction, and neocolonial structures (Cameron 2012; Hanaček et al. 2022).

A political ecology lens offers more than a critique of inequality—it helps reveal how adaptation can become stratified, benefiting some while excluding others, and how interventions risk reinforcing inequalities if structural determinants are not addressed (Sovacool 2018b; Hendricks and Van Zandt 2021; Malik and Ford 2025c; Taylor 2014). By making these dynamics visible, political ecology urges a shift from superficial resilience-building toward redistributive, justice-oriented adaptation. This means centring Indigenous governance in policy decisions, ensuring sustained investment in local infrastructure, and recognising Inuit knowledge systems not as complementary but as essential (Whyte 2017; Nadasdy 2003; Smith 2021; Malik et al. 2025b). More broadly, applying this lens in other Arctic regions can help uncover the embedded hierarchies within adaptation programming and promote place-based, historically informed, and community-led responses. Without addressing these underlying drivers of vulnerability, adaptation risks becoming another site of exclusion and inequity. As climate impacts intensify, integrating this perspective is important for crafting responses that are not only effective, but also equitable and decolonial.

## Conclusion

To build a more resilient future for Nunatsiavut, it is essential to address the root causes of inequity and systemic barriers that limit effective adaptation, particularly for marginalised groups. This includes addressing the high costs of living, the economic burdens of technological dependence, and the infrastructural challenges that exacerbate vulnerabilities. An increase in the deployment of freight ships should be done to ensure the consistent delivery of food supplies. Healthcare access should be enhanced through greater presence of healthcare professionals, the establishment of more community health clinics, and the expansion of mental health services. Improving housing conditions, providing subsidies for food, and providing financial assistance are necessary to mitigate the high cost of living. Implementing subsidies for new, fuel-efficient stoves is important to promote energy efficiency and reduce environmental impact. Increasing the number of flights, along with reducing airfare costs, is important to ensure reliable transportation and facilitation of timely medical appointments and other vital services. Creating job opportunities for communities, promoting local food production, and active participation of Inuit in decision-making processes are important in addressing adaptation challenges.

## Data Availability

All data is available within the manuscript.
